# Supplementary figures and images for: Effects of organic fertilizer on soil nutrient status, enzyme activity, and bacterial community diversity in Leymus chinensis steppe in Inner Mongolia, China
Source: PLoS One. 2020 Oct 15;15(10):e0240559. doi: 10.1371/journal.pone.0240559 (PMC7561123; doi:10.1371/journal.pone.0240559)

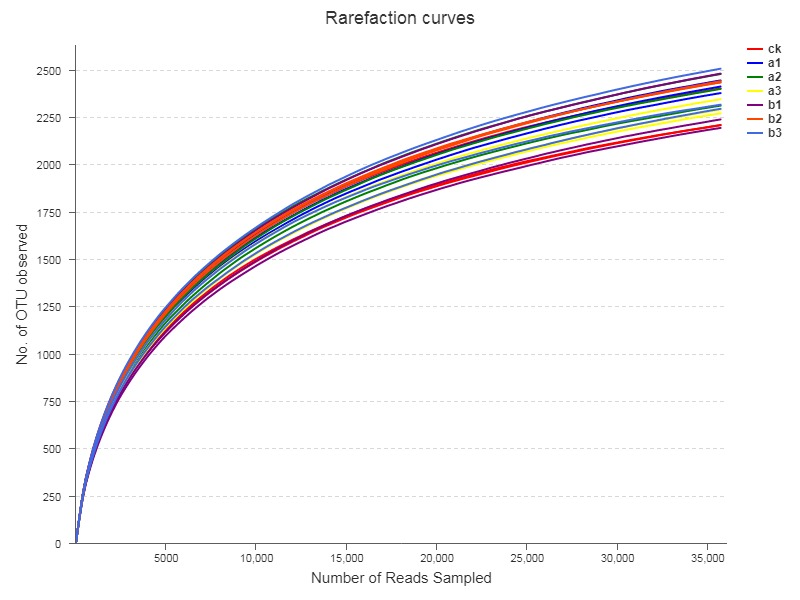

Supplement: S1 Fig — ck, no fertilizer application treatment; a1, a2, a3, vermicompost fertilizer treatments; b1, b2, b3, mushroom residue fertilizer treatments. (TIF) [file pone.0240559.s001.tif]
