# Supplementary material for: Effects of organic fertilizer on soil nutrient status, enzyme activity, and bacterial community diversity in Leymus chinensis steppe in Inner Mongolia, China
Source: PLoS One. 2020 Oct 15;15(10):e0240559. doi: 10.1371/journal.pone.0240559 (PMC7561123; doi:10.1371/journal.pone.0240559)
Supplement: S1 Table — (DOCX) [file pone.0240559.s002.docx]

**Table S1 Sequencing data and OTUs across treatments.**

| Treatment | Raw paired reads | Quality filtered reads | No. of OTUs |
| --- | --- | --- | --- |
| Ick | 70798 | 53884 | 2208 |
| IIck | 67263 | 43698 | 2402 |
| IIIck | 62044 | 39480 | 2208 |
| Ia1 | 63283 | 43830 | 2444 |
| IIa1 | 66290 | 45938 | 2377 |
| IIIa1 | 74968 | 48523 | 2412 |
| Ia2 | 68860 | 46632 | 2311 |
| IIa2 | 73278 | 49316 | 2478 |
| IIIa2 | 67847 | 49286 | 2399 |
| Ia3 | 66419 | 44529 | 2270 |
| IIa3 | 69624 | 49046 | 2290 |
| IIIa3 | 65239 | 41989 | 2345 |
| Ib1 | 63248 | 41570 | 2238 |
| IIb1 | 73105 | 52317 | 2193 |
| IIIb1 | 61695 | 40042 | 2480 |
| Ib2 | 57793 | 42023 | 2435 |
| IIb2 | 71002 | 48625 | 2440 |
| IIIb2 | 53939 | 35708 | 2433 |
| Ib3 | 68023 | 45676 | 2316 |
| IIb3 | 71051 | 46611 | 2294 |
| IIIb3 | 69683 | 46478 | 2506 |
| Raw paired reads, raw sequencing data; quality filtered reads, clean data after filtering and removal of chimerical sequences, singletons, replicates, and chimeras; number of operational taxonomic units (OTUs), generated by clustering quality filtered reads based on 97% similarity. ck, no fertilization treatment; a1, a2, a3, vermicompost fertilizer treatments; b1, b2, b3, mushroom residue fertilizer treatments; I, II, and III indicate replicates. | | | |


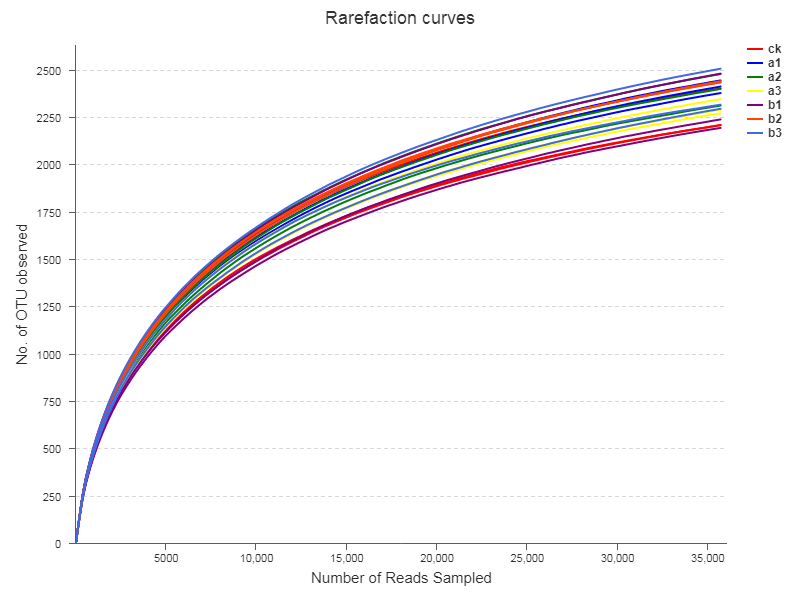


**Figure S1 Rarefaction curves for each sample.** ck, no fertilization treatment; a1, a2, a3, vermicompost fertilizer treatments; b1, b2, b3, mushroom residue fertilizer treatments.
