# Supplementary material for: Effects of organic fertilizer on soil nutrient status, enzyme activity, and bacterial community diversity in Leymus chinensis steppe in Inner Mongolia, China
Source: PLoS One. 2020 Oct 15;15(10):e0240559. doi: 10.1371/journal.pone.0240559 (PMC7561123; doi:10.1371/journal.pone.0240559)
Supplement: S2 Table — The results of a permutation test of environmental variables and soil samples based on the bacterial 16s rRNA data at the operational taxonomic unit (OTU) level. The p values were based on 999 permutations. (DOCX) [file pone.0240559.s003.docx]

**Table S2** **Correlation between 16s rRNA data of bacteria treated with organic fertilizer and environmental factors.** The results of a permutation test of environmental variables and soil samples based on the bacterial 16s rRNA data at the operational taxonomic unit (OTU) level. The p values were based on 999 permutations.

| Environmental variables | RDA1 | RDA2 | r2 | P_values |
| --- | --- | --- | --- | --- |
| TP | 0.0185 | 0.9998 | 0.3076 | 0.032 |
| TK | –0.9839 | –0.1785 | 0.0293 | 0.777 |
| AN | –0.8109 | 0.5853 | 0.0927 | 0.43 |
| AP | 0.9187 | 0.3949 | 0.0063 | 0.954 |
| AK | –0.5415 | 0.8407 | 0.0528 | 0.65 |
| SOM | –0.8401 | 0.5424 | 0.0132 | 0.902 |
| S_UE | –0.1596 | –0.9872 | 0.2938 | 0.044 |
| S_SC | –0.7366 | –0.6764 | 0.0028 | 0.974 |
| S_AKP | 0.2366 | 0.9716 | 0.1792 | 0.172 |
| S_CAT | 0.2653 | 0.9642 | 0.1215 | 0.301 |
| DM | –0.4109 | –0.9117 | 0.0988 | 0.415 |
