# Supplementary material for: Effects of organic fertilizer on soil nutrient status, enzyme activity, and bacterial community diversity in Leymus chinensis steppe in Inner Mongolia, China
Source: PLoS One. 2020 Oct 15;15(10):e0240559. doi: 10.1371/journal.pone.0240559 (PMC7561123; doi:10.1371/journal.pone.0240559)
Supplement: Attachment — Submitted filename: Response to Reviewers.docx [file pone.0240559.s004.docx]

Response to Reviewer 1 Comments

Reviewer #1: Comments:

1) Line no 24: “that” is repeated

**Response 1):** We thank the reviewer for the time in closely viewing our manuscript. Done.

2) Line no 55: put a full stop after “processes”

**Response 2):** It has been modified in the revised version..

3) Line no 56: use the term “microbial” instead of “microbe”

**Response 3):** Done. The sentence has been revised as suggested by the reviewer.

4) Line no 55-57: “Because of……..after fertilization”, provide some references of similar studies

**Response 4):** The reviewer’s suggestion has been adopted and some similar references have been cited in the revision.

5) Line no 60: Instead of “studies of the” use “studies on the”

**Response 5):** It has been modified in the revised version.

6) Line no 68-70: use either of the two terms “were conducted” or “was conducted”

**Response 6):** Modified in the revised version. Use “was conducted”.

7) Line no 193: use another term in place of “dramatically”

**Response 7):** The reviewer’s suggestions have been adopted. It has been modified in the revised version. Use “significantly”. The application of vermicompost and mushroom residue organic fertilizers significantly altered soil chemical properties (Table 1).

8) Line no 356: mention reference number for Chu et al.

**Response 8):** Added chu et al references in the revised version.

9) Line no 195-197: The percentage increase in soil parameters viz. TP and TK mentioned in the result section is not significant as compared to control values. Only significant results should be mentioned in the result section supported by appropriate references in the discussion part.

**Response 9):** The reviewer’s suggestion has been adopted and only significant results should be mentioned in the result section supported by appropriate references in the discussion part. Corresponding references have been added to the discussion section of the revised version. Following the application of organic manure, Chu et al [32] found that SOC and the major soil nutrients (N, P, and K) were also significantly increased in a sandy loam soil. Zhen et al. [33] experiment results showed that long-term fertilization influenced soil properties and microbial community. These changes in soil properties can be attributed to the variation in soil microbial community after fertilizer application.

10) Line no 199-200: The percentage increase in soil parameters viz. AN and SOM is again not significant in comparison to control values.

**Response 10):** The reviewer’s suggestion has been adopted and this part has been modified in the revised version. Soil AN and SOM were not significantly affected by vermicompost and mushroom residue applications.

11) Line no 201-202: In addition to mentioned parameters (TN, AN & SOM), TP, TK and AP (except for treatment a3) also didn’t show significant variations and should be mentioned.

**Response 11):** The reviewer’s suggestion has been adopted and this part has been modified in the revised version.

12) Line no 456-457-458: Provide some references.

**Response 12):** It has been modified in the revised version and added corresponding references.

13) Provide some recent references of current year.

**Response 13):** The reviewer’s suggestion has been adopted and an updated reference has been cited in the revision.

14) The discussion section can be further strengthened by citing more similar studies.

**Response 14):** The reviewer’s suggestions have been adopted and the discussion section has been revised, and corresponding similar documents have been added.

Response to Reviewer 2 Comments

Reviewer #2: The research work has been done in intelligent manner. Data has been put with sound statistical analysis. However certain point need to be corrected as:

1. The term 'fertilization' often used in manuscript which should be replaced with 'fertilizer application'

**Response 1:** We thank the reviewer for the time in closely viewing our manuscript. The "fertilization" in the article has been changed to "fertilizer application".

2. few reference nos are not correct which are highlighted/corrected

**Response 2:** Done. The references of the manuscript have been checked and revised in the revised version.

3. Discussions particularly related to sequencing of microbial community diversity need more clear presentation.

**Response 3:** The reviewer’s suggestion has been adopted. We have revised the paper based on the reviewer’s comments and discussion related to sequencing of microbial community diversity has been modified.
